# Intrathoracic hemocoagulase *Bothrops atrox* injection efficacy in thoracoscopic lung cancer surgery

**DOI:** 10.1097/MD.0000000000046001

**Published:** 2025-11-14

**Authors:** Yilian Xie, Jianwei Han, Jiajia Yang, Wei Xiong, Junsen Ye, Aiming Yang, Yingding Ruan

**Affiliations:** a Department of Pharmacy, The First People’s Hospital of Jiande, Jiande, China; b Department of Thoracic Surgery, The First People’s Hospital of Jiande, Jiande, China; c Department of Scientific Research and Education, The First People’s Hospital of Jiande, Jiande, China.

**Keywords:** coagulation profiles, hemocoagulase *Bothrops atrox*, intrathoracic injection, lung cancer, video-assisted thoracoscopic surgery

## Abstract

Hemocoagulase *Bothrops atrox* (HBA) has been widely used to reduce perioperative bleeding in surgical procedures, while little is known about its specific efficacy and safety in video-assisted thoracoscopic surgery (VATS). This study aimed to evaluate the impact of intrathoracic HBA injection on perioperative outcomes and coagulation in patients undergoing VATS. We retrospectively included 388 patients who had lung cancer and received VATS. They were categorized into HBA (n = 51) and non-HBA (n = 337) groups. After 1:2 propensity score matching, 120 patients (45 HBA and 75 non-HBA) were included in final analysis. Multivariate analysis was performed to identify independent factors influencing postoperative thrombin time (TT). Before propensity score matching, the HBA group had significantly lower intraoperative bleeding volume, surgery duration, postoperative hospital stay, drainage volume, and drainage time compared to the non-HBA group (*P* < .001). Additionally, the HBA group had higher rates of comorbidities, more postoperative complications, and worse pre- and postoperative coagulation functions evaluated by international standardized ratio, activated partial thrombin time, prothrombin time, and fibrinogen level. After matching, the HBA group still demonstrated a higher TT (*P* = .006), shorter postoperative hospital stays (*P* = .001), and a lower drainage volume (*P* = .003) compared to non-HBA group. Multivariate analysis revealed that HBA (*P* = .009) and preoperative TT (*P* < .001) were independent risk factors for prolonged postoperative TT. Our study revealed that intrathoracic HBA injection in VATS may reduce postoperative hospital stay and drainage volume, albeit with prolonged TT. Monitoring coagulation function before and after surgery is necessary for patients receiving HBA during surgery.

## 1. Introduction

Lung cancer is the leading cause of cancer incidence and mortality worldwide, with thoracic surgery remaining to be the irreplaceable treatment, especially for early-stage non-small cell lung cancer (NSCLC).^[1,2]^ The development of surgical techniques, such as video-assisted thoracoscopic surgery (VATS), has significantly improved patient outcomes by minimizing surgical trauma, reducing postoperative pain, and accelerating recovery.^[3]^ Nonetheless, perioperative bleeding remains a threatening issue that leads to higher morbidity, extended hospital stays, and increased medical costs.^[4]^

To effectively manage intraoperative bleeding, hemocoagulase *Bothrops atrox* (HBA) is widely used in various surgical procedures in recent years due to its potent hemostatic properties. However, there remains a lack of systematic and comprehensive research characterizing the effects of intrapleural HBA injection during VATS on patient’s outcomes including postoperative coagulation profile, postoperative drainage, hospital stays, and postoperative complications. Herein, this retrospective study aims to evaluate the efficacy and safety of intrapleural HBA injection in patients who had lung cancer and received VATS treatment.

## 2. Methods

### 2.1. Study design

This retrospective study primarily included patients who underwent VATS at our hospital between December 1, 2021 and July 31, 2024. We obtained these data between August 2, 2024 and August 28, 2024. The surgery was performed by the same thoracic surgical team throughout the study. The inclusion criteria for patients were: patients received VATS and had a postoperative pathological diagnosis of NSCLC. Exclusion criteria included: previous chest surgery; benign tumor revealed by pathology; non-primary lung tumor; preoperative radiotherapy, chemotherapy, immunotherapy, targeted therapy, or other treatment; consecutive surgery within 1 month; transfer to another hospital; stage IV or palliative surgery; non-NSCLC; history of hepatitis B, cirrhosis, and splenectomy; loss of coagulation function test within 1 day after surgery.

The tumor, node, and metastasis classification is determined according to the International Association for the Study of Lung Cancer.^[5]^

This study complied with the Declaration of Helsinki and was approved by the Ethics Committee of The First People’s Hospital of Jiande (Ethics Committee approval number: 20240805004). The informed consent was waived because of the retrospective nature of the study.

### 2.2. Hemocoagulase *Bothrops atrox*

HBA, also known as Batroxobin, is a thrombin-like and coagulation kinase-like enzyme isolated from the venom of the Brazilian spearhead pit viper.^[6]^ It exhibits potent antifibrinolytic properties by cleaving fibrinogen’s α-chains, releasing fibrinopeptides A and B, and accelerating the formation of a stable fibrin clot.^[7–10]^ Therefore, HBA effectively achieve hemostasis. Importantly, HBA has no effects on uninjured blood vessels, so that it is exempted from increasing the risk of intraoperative or postoperative thrombosis and disseminated intravascular coagulation.^[8,11–13]^

### 2.3. Procedures of HBA administration

A total of 51 patients received intrathoracic HBA injection during VATS. Briefly, after the completion of lung resection but before closure, 10 units of HBA were mixed with 5 mL 0.9% normal saline and injected into the surgical wound of the pleural cavity under direct visualization via VATS, using a sterile syringe connected to a long intravenous tubing.

### 2.4. Data collection

Patient demographics, clinicopathologic features, and operation details were retrospectively collected. This included sex, age, body mass index, smoking history, comorbidities (hypertension, diabetes, coronary heart disease, and chronic obstructive pulmonary disease), circulatory stimulants, surgical approach, tumor, node, and metastasis stage, resection site and type, lymph node retrieval and stations explored, surgical duration, intraoperative bleeding, drainage time and volume, postoperative hospital stay, postoperative complications, pathological types, tumor size, and imaging description (ground glass nodule, mixed nodule, or solid nodule). Preoperative and postoperative albumin and coagulation function, including D-dimer, international standardized ratio (INR), activated partial thrombin time (APTT), thrombin time (TT), prothrombin time (PT), and fibrinogen (FIB) were also recorded. Blood samples for coagulation testing were obtained 3 days before surgery and 1 day after surgery.

### 2.5. Statistical analysis

To minimize potential bias, a propensity score matching (PSM) analysis was conducted with a 1:2 ratio and a caliper value of 0.4. Those variables with a liberal threshold of *P* < .05 were included in the propensity model to ensure their clinical relevance and statistical discernment. The propensity scores were calculated using a logistic analysis incorporating surgical approach, type of lung resection, intraoperative bleeding volume, surgical duration, as well as preoperative coagulation indices including INR, APTT, and PT, all of which are known to potentially influence the surgical outcome.

Data were presented as mean ± SD or median (P25, P75). Normally distributed data were analyzed using Student *t* test, while non-normally distributed data were assessed with the Wilcoxon rank-sum test. Categorical data were expressed as frequency (%) and analyzed using Chi-square or Fisher exact test. Variables with *P* < .05 in univariate logistic regression analysis was subjected to multivariate analysis to identify independent factors. All statistical analyses were performed using SPSS version 22.0 (IBM Corp., Armonk) and *P* < .05 was considered as statistically significant.

## 3. Results

### 3.1. Characteristics of patients

**Figure 1.**
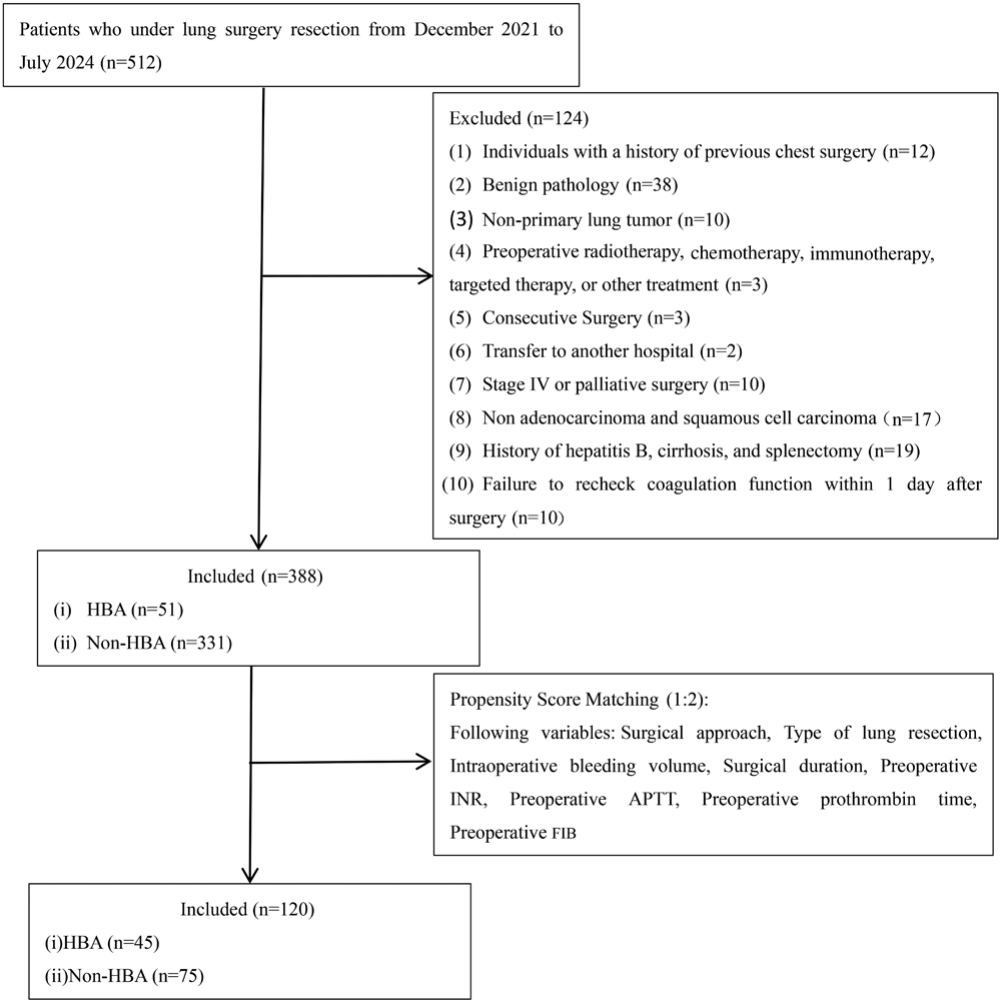
Flowchart of patient selection. APTT = activated partial thrombin time, FIB = fibrinogen, HBA = hemocoagulase *Bothrops atrox*, INR = international standardized ratio.

The flowchart of patient selection is shown in Figure 1. Initially, we enrolled 512 patients who had NSCLC and received VATS in our hospital from December 2021 to July 2024. After applying inclusion and exclusion criteria, 388 were included in the study. After PSM, 120 patients were included in the analysis: 45 (37.5%) in HBA group and 75 (62.5%) in non-HBA group; 54 males (45%) and 66 females (55%); mean age, 61.83 ± 11.98 years (Table 1).

**Table 1:** Patient characteristics and treatment status before and after PSM (n [%], mean ± SD, *M* [P25, P75]).

Variables	Before propensity matching	After propensity matching
	Number (n = 388)	Number (n = 120)
HBA, n (%)	51 (13.1)	45 (37.5)
Sex, n (%)		
Male	174 (44.9)	54 (45)
Female	214 (55.1)	66 (55)
Smoking, n (%)	116 (29.9)	37 (30.8)
Comorbidities, n (%)	150 (38.7)	31 (25.8)
Circulatory stimulants, n (%)	10 (2.6)	4 (3.3)
Age (mean ± SD)	62.92 ± 10.48	61.83 ± 11.98
BMI (mean ± SD)	22.75 ± 3.49	22.51 ± 3.55
Pathological types, n (%)		
Adenocarcinoma	344 (88.7)	104 (86.7)
Squamous cell carcinoma	44 (11.3)	16 (13.3)
TNM stage, n (%)		
IA/B	360 (92.8)	115 (95.8)
IIA/B	12 (3.1)	2 (1.7)
IIIA/B	16 (4.1)	3 (2.5)
Surgical approach, n (%)		
U-VATS	232 (59.8)	90 (75)
M-VATS	156 (40.2)	30 (25)
Tumor size, n (%)		
≤3 cm	360 (92.8)	110 (91.7)
3–5 cm	15 (3.9)	6 (5)
>5 cm	13 (3.3)	4 (3)
Imaging description, n (%)		
Ground glass nodule	96 (24.7)	33 (27.5)
Mixed nodule	134 (34.6)	36 (30)
Solid nodule	158 (40.7)	51 (42.5)
Position, n (%)		
Left	166 (42.8)	62 (51.7)
Right	222 (57.2)	58 (48.3)
Resection site, n (%)		
Right upper	121 (31.2)	33 (27.5)
Right middle	26 (6.7)	5 (4.2)
Right lower	73 (18.8)	20 (16.7)
Left upper	97 (25)	34 (28.3)
Left lower	71 (18.3)	28 (23.3)
Type of lung resection, n (%)		
Lobectomy	219 (56.4)	38 (31.7)
Segmental	80 (20.6)	26 (21.7)
Wedge	89 (23)	56 (46.6)
Intraoperative bleeding volume (*M* [P25, P75])	100 (50, 100)	50 (20, 50)
Surgical duration (*M* [P25, P75])	125 (90, 168)	90 (60.75, 120)
Number of mediastinal lymph nodes retrieved (*M* [P25, P75])	4 (0, 9.25)	1 (0, 5)
Mediastinal lymph node stations explored (*M* [P25, P75])	2 (0, 3)	1 (0, 3)
Drainage volume (*M* [P25, P75])	750 (417.5, 1200)	590 (250, 927.5)
Drainage time (*M* [P25, P75])	5 (3, 9)	4 (3, 5)
Postoperative complications, n (%)	12 (3.1)	6 (5)
Postoperative hospital stay (*M* [P25, P75])	8.40 (5.4, 11.41)	6 (4, 8)
Preoperative albumin level (mean ± SD)	43.22 ± 4.50	43.35 ± 4.48
Preoperative D-dimer (*M* [P25, P75])	0.27 (0.17, 0.49)	0.25 (0.17, 0.37)
Preoperative INR (mean ± SD)	0.97 ± 0.10	1.01 ± 0.13
Preoperative APTT (mean ± SD)	27.82 ± 3.78	29.48 ± 3.72
Preoperative TT (mean ± SD)	17.90 ± 2.01	17.64 ± 2.04
Preoperative PT (mean ± SD)	11.39 ± 1.14	11.87 ± 1.44
Preoperative FIB (*M* [P25, P75])	277.8 (236, 319.8)	284.7 (247.83, 331.95)
Postoperative albumin level (mean ± SD)	34.08 ± 4.08	34.98 ± 3.91
Postoperative D-dimer (*M* [P25, P75])	1.39 (0.80, 2.95)	1.27 (0.78, 2.43)
Postoperative INR (mean ± SD)	1.07 ± 0.41	1.1 ± 0.43
Postoperative APTT (mean ± SD)	30.49 ± 4.76	31.86 ± 4.64
Postoperative TT (mean ± SD)	16.50 ± 1.92	16.27 ± 1.74
Postoperative PT (mean ± SD)	12.26 ± 1.50	12.68 ± 1.47
Postoperative FIB (*M* [P25, P75])	310.34 (263.85, 392.3)	304.9 (267.3, 390.03)

APTT = activated partial thrombin time, BMI = body mass index, FIB = fibrinogen, HBA = hemocoagulase *Bothrops atrox*, INR = international standardized ratio, *M* (P25, P75) = median (25th percentile,75th percentile), M-VATS = multiportal video-assisted thoracoscopic surgery, PSM = propensity score matching, PT = prothrombin time, TNM = tumor, node, and metastasis, TT = thrombin time, U-VATS = uniportal video-assisted thoracoscopic surgery.

PSM effectively eliminated confounding factors (Table 2). Compared to the non-HBA group, the HBA group exhibited a significantly higher postoperative TT (16.83 ± 1.99 vs 15.94 ± 1.49, *P* = .006), a shorter postoperative hospital stay (6 vs 5 days, *P* = .001), and lower drainage volumes (400 [200, 700] vs 650 [350, 1072.5] mL, *P* = .033).

**Table 2:** Comparison and statistical analysis of patient characteristics before and after PSM (n [%], mean ± SD, *M* [P25, P75]).

Variables	Before propensity matching			After propensity matching		
	Non-HBA (n = 337)	HBA (n = 51)	*P*	HBA (n = 45)	Non-HBA (n = 75)	*P*
Sex, n (%)			.792			.925
Male	152 (45.1)	22 (43.1)		20 (44.4)	34 (45.3)	
Female	185 (54.9)	29 (56.9)		25 (55.6)	41 (54.7)	
Smoking, n (%)	100 (29.7)	16 (31.4)	.805	14 (31.111)	23 (30.667)	.959
Comorbidities, n (%)	139 (41.3)	11 (21.6)	.007	11 (24.4)	20 (26.7)	.788
Circulatory stimulants, n (%)	7 (2.1)	3 (5.9)	.261	3 (6.7)	1 (1.3)	.294
Age (mean ± SD)	62.93 ± 10.24	62.80 ± 12.05	.934	63.53 ± 11.42	60.8 ± 12.26	.228
BMI (mean ± SD)	22.82 ± 3.52	22.31 ± 3.29	.331	22.38 ± 3.41	22.59 ± 3.64	.749
Pathological types, n (%)			.564			1.000
Adenocarcinoma	300 (89)	44 (86.23)		39 (86.7)	65 (86.7)	
Squamous cell carcinoma	37 (11)	7 (13.7)		6 (13.3)	10 (13.3)	
TNM stage, n (%)			.327			.913
IA/B	311 (92.3)	49 (96)		43 (95.6)	72 (96)	
IIA/B	11 (3.3)	1 (2)		1 (2.2)	1 (1.3)	
IIIA/B	15 (4.4)	1 (2)		1 (2.2)	2 (2.7)	
Surgical approach, n (%)			.001			.586
U-VATS	191 (56.7)	41 (80.4)		35 (77.8)	55 (73.3)	
M-VATS	146 (43.3)	10 (19.6)		10 (22.2)	20 (26.7)	
Tumor size, n (%)			.914			.937
≤3 cm	313 (92.9)	47 (92.2)		41 (91.111)	69 (92.000)	
3–5 cm	11 (3.2)	4 (7.8)		4 (8.889)	2 (2.667)	
>5 cm	13 (3.9)	0 (0)		0 (0.000)	4 (5.333)	
Imaging description, n (%)			.643			.883
Ground glass nodule	17 (33.3)	79 (23.4)		14 (31.1)	19 (25.3)	
Mixed nodule	12 (23.5)	122 (36.2)		10 (22.2)	26 (34.7)	
Solid nodule	22 (43.2)	136 (40.4)		21 (46.7)	30 (40)	
Position, n (%)			.204			.777
Left	140 (41.5)	26 (51)		24 (53.3)	38 (50.7)	
Right	197 (58.5)	25 (49)		21 (46.7)	37 (49.3)	
Resection site, n (%)			.624			.784
Right upper	103 (30.6)	18 (35.3)		16 (35.6)	17 (22.7)	
Right middle	22 (6.5)	4 (7.8)		2 (4.4)	3 (4)	
Right lower	70 (20.8)	3 (5.9)		3 (6.7)	17 (22.7)	
Left upper	84 (24.9)	13 (25.5)		12 (26.7)	22 (29.3)	
Left lower	58 (17.2)	13 (25.5)		12 (26.7)	16 (21.3)	
Type of lung resection, n (%)			<.001			.673
Lobectomy	207 (61.4)	12 (23.5)		12 (26.7)	26 (34.7)	
Segmental	68 (20.2)	12 (23.5)		12 (26.7)	14 (18.7)	
Wedge	62 (18.4)	27 (53)		21 (46.6)	35 (46.6)	
Intraoperative bleeding volume (*M* [P25, P75])	100 (50, 100)	50 (20, 50)	<.001	50 (20, 50)	50 (20, 50)	.961
Surgical duration (*M* [P25, P75])	132 (96, 175)	90 (60, 110)	<.001	90 (60, 110)	92 (62, 121)	.501
Number of mediastinal lymph nodes retrieved (*M* [P25, P75])	2 (1, 4.5)	4 (0, 10)	.219	2 (1, 5)	1 (0, 5)	.116
Mediastinal lymph node stations explored (*M* [P25, P75])	2 (1, 3)	2 (0, 3)	.89	1. (1, 3)	1 (0, 3)	.105
Drainage volume (*M* [P25, P75])	810 (500, 1270)	400 (185, 605)	<.001	400 (200, 700.)	650 (350, 1072.5)	.003
Drainage time (*M* [P25, P75])	5 (3.12, 9)	3 (2, 4)	<.001	3 (3, 4)	4 (3, 6)	.067
Postoperative complications, n (%)	10 (3.0)	2 (3.9)	.714	2 (4.4)	4 (5.3)	.829
Postoperative hospital stay (*M* [P25, P75])	9 (6.38, 12.38)	4 (3.5, 6)	<.001	5 (4, 6)	6 (5, 9)	.001
Preoperative albumin level (mean ± SD)	43.24 ± 4.57	43.10 ± 4.00	.836	43.27 ± 3.426	43.39 ± 5.025	.882
Preoperative D-dimer (*M* [P25, P75])	0.28 (0.16, 0.53)	0.25 (0.20, 0.33)	.86	0.27 (0.2, 0.33)	0.21 (0.14, 0.45)	.304
Preoperative INR (mean ± SD)	0.95 ± 0.09	1.03 ± 0.16	.001	1.03 ± 0.17	1.00 ± 0.10	.366
Preoperative APTT (mean ± SD)	27.49 ± 3.70	29.95 ± 3.58	<.001	29.82 ± 3.6	29.27 ± 3.79	.427
Preoperative TT (mean ± SD)	17.88 ± 1.97	18.03 ± 2.30	.63	17.89 ± 2.34	17.48 ± 1.83	.292
Preoperative PT (mean ± SD)	11.28 ± 0.98	12.07 ± 1.77	<.001	11.99 ± 1.86	11.8 ± 1.11	.499
Preoperative FIB (*M* [P25, P75])	273.7 (232.3319.15)	290.4 (267.8, 340.1)	.051	290.8 (271.05, 341.20)	281.8 (236, 329.1)	.375
Postoperative albumin level (mean ± SD)	35.65 ± 3.02	33.84 ± 4.17	<.001	34.79 ± 3.78	34.49 ± 4.4	.358
Postoperative D-dimer (mean ± SD)	1.22 (0.81, 2.06)	1.42 (0.80, 2.98)	.448	1.25 (0.91, 2.09)	1.39 (0.74, 3.01)	.826
Postoperative INR (mean ± SD)	1.11 ± 0.11	1.07 ± 0.44	.36	1.11 ± 0.02	1.1 ± 0.06	.064
Postoperative APTT (mean ± SD)	30.15 ± 4.73	32.72 ± 4.36	<.001	32.48 ± 4.2	31.49 ± 4.87	.26
Postoperative TT (mean ± SD)	16.43 ± 1.88	16.90 ± 2.11	.103	16.83 ± 1.99	15.94 ± 1.49	.006
Postoperative PT (mean ± SD)	12.15 ± 1.50	12.98 ± 1.28	<.001	12.93 ± 1.32	12.54 ± 1.54	.158
Postoperative FIB (*M* [P25, P75])	319.10 (260.20, 396.70)	300.50 (266.45, 365.80)	.341	300.5 (279.8, 355)	304.9 (264.1, 399.8)	.353

APTT = activated partial thrombin time, BMI = body mass index, FIB = fibrinogen, HBA = hemocoagulase *Bothrops atrox*, INR = international standardized ratio, *M* (P25,P75) = median (25th percentile,75th percentile), M-VATS = multiportal video-assisted thoracoscopic surgery, PSM = propensity score matching, PT = prothrombin time, TNM = tumor, node, and metastasis, TT = thrombin time, U-VATS = uniportal video-assisted thoracoscopic surgery.

### 3.2. Postoperative complications

Before PSM, the major postoperative complications included lung infections in 6 patients, prolonged air leaks after surgery in 3 patients, poor wound healing in 1 patient, and postoperative delirium in 2 patients. After PSM, 2 patients had lung infections in HBA group, while the non-HBA group had 4 patients with lung infections and 1 patient with prolonged air leaks after surgery. There were no deaths during hospitalization and 30 days after discharge and no patients experienced postoperative bleeding or thrombosis after surgery.

### 3.3. Subgroup analysis of postoperative TT

PSM analysis revealed a significant association between intrathoracic HBA injection and postoperative TT. To delve into the potential influence of other factors on postoperative TT, we conducted both univariate and multivariate logistic regression analyses. The univariate analysis indicated that HBA (*P* = .006), preoperative TT (*P* < .001), and preoperative PT (*P* = .041) were associated with prolonged postoperative TT. The subsequent multivariate analysis further confirmed that HBA (*P* = .009) and preoperative TT (*P* < .001) were independent factors for prolonged postoperative TT (Table 3).

**Table 3:** Results of univariate and multivariable logistic regression analyses for postoperative TT.

Variables	Univariate logistic regression analyses						Multivariable logistic regression analyses				
	β	SE	*t*	*P*	β (95% CI)	β		SE	*t*	*P*	β (95%CI)
HBA	0.89	0.32	2.78	.006	0.89 (0.26 to 1.52)	0.76		0.29	2.65	.009	0.76 (0.20 to 1.32)
Sex											
Male					0.00 (Reference)						
Femal	0.08	0.32	0.25	.799	0.08 (−0.55 to 0.71)						
Pathological types											
Adenocarcinoma					0.00 (Reference)						
Squamous cell carcinoma	0.29	0.47	0.61	.543	0.29 (−0.63 to 1.20)						
TNM stage											
IA/B					0.00 (Reference)						
IIA/B	0.94	1.25	0.75	.454	0.94 (−1.51 to 3.39)						
IIIA/B	−0.26	1.02	−0.26	.799	−0.26 (−2.27 to 1.75)						
Tumor size											
≤3 cm					0.00 (Reference)						
3–5cm	0.17	0.74	0.24	.813	0.17 (−1.27 to 1.62)						
>5 cm	0.09	0.89	0.1	.919	0.09 (−1.66 to 1.84)						
Imaging description											
Ground glass nodule					0.00 (Reference)						
Mixed nodule	−0.01	0.42	−0.01	.99	−0.01 (−0.83 to 0.82)						
Solid nodule	0.18	0.39	0.47	.639	0.18 (−0.58 to 0.95)						
Position											
Left					0.00 (Reference)						
Right	−0.11	0.32	−0.36	.722	−0.11 (−0.74 to 0.51)						
Lobectomy											
Right upper					0.00 (Reference)						
Right middle	−0.52	0.85	−0.62	.537	−0.52 (−2.19 to 1.14)						
Right lower	−0.08	0.5	−0.16	.874	−0.08 (−1.06 to 0.90)						
Left upper	0.03	0.43	0.06	.95	0.03 (−0.82 to 0.87)						
Left lower	0.06	0.45	0.13	.899	0.06 (−0.83 to 0.95)						
Type of lung resection											
Lobectomy					0.00 (Reference)						
Segmental	0	0.44	0.01	.991	0.00 (−0.86 to 0.87)						
Wedge	−0.41	0.37	−1.12	.267	−0.41 (−1.13 to 0.31)						
Surgical approach											
U-VATS					0.00 (Reference)						
M-VATS	0.63	0.36	1.74	.084	0.63 (−0.08 to 1.35)						
Comorbidities	−0.1	0.36	−0.26	.794	−0.10 (−0.81 to 0.62)						
Circulatory stimulants	−0.36	0.89	−0.4	.688	−0.36 (−2.10 to 1.38)						
Smoking	0.01	0.35	0.02	.984	0.01 (−0.67 to 0.68)						
Age	0.02	0.01	1.45	.151	0.02 (−0.01 to 0.05)						
BMI	−0.07	0.04	−1.47	.144	−0.07 (−0.15 to 0.02)						
Number of mediastinal lymph nodes retrieved	0.05	0.04	1.28	.204	0.05 (−0.03 to 0.12)						
Mediastinal lymph node stations explored	0.09	0.09	1.03	.303	0.09 (−0.08 to 0.27)						
Intraoperative bleeding volume	0	0	−0.57	.569	−0.00 (−0.00 to 0.00)						
Duration of surgery	0	0	−0.14	.893	−0.00 (−0.01 to 0.01)						
Preoperative albumin level	−0.02	0.04	−0.64	.523	−0.02 (−0.09 to 0.05)						
Preoperative D-dimer	−0.08	0.15	−0.53	.597	−0.08 (−0.37 to 0.21)						
Preoperative INR	−2.04	1.21	−1.69	.094	−2.04 (−4.40 to 0.33)						
Preoperative APTT	−0.04	0.04	−1.03	.304	−0.04 (−0.13 to 0.04)						
Preoperative TT	0.41	0.07	5.85	<.001	0.41 (0.27 to 0.54)	0.37		0.07	5.21	<.001	0.37 (0.23 to 0.51)
Preoperative PT	−0.23	0.11	−2.07	.041	−0.23 (−0.44 to −0.01)	−0.1		0.1	−1.04	.301	−0.10 (−0.30 to 0.09)
Preoperative FIB	0	0	−1.69	.093	−0.00 (−0.01 to 0.00)						

APTT = activated partial thrombin time, BMI = body mass index, CI = confidence interval, FIB = fibrinogen, HBA = hemocoagulase *Bothrops atrox*, INR = international standardized ratio, M-VATS = multiportal video-assisted thoracoscopic surgery, PT = prothrombin time, SE = standard error, TNM = tumor, node, and metastasis, TT = thrombin time, U-VATS = uniportal video-assisted thoracoscopic surgery.

## 4. Discussion

This study examined the effect of intrathoracic HBA injection on patient’s outcome after VATS. Our results indicated that HBA injection significantly increased postoperative TT, demonstrating its efficacy in maintaining hemostasis after surgery. Meanwhile, patients in HBA group also had shorter hospital stays and less drainage volume compared to non-HBA group, indicating a favorable outcome. Preoperative TT and HBA independently affected postoperative TT.

HBA has been widely utilized in surgical procedures due to its hemostatic potency in clinical practice.^[11,12,14–24]^ Gupta et al^[15]^ compared topical thrombin use and saline compression dressings in tooth extraction. They found that the thrombin group significantly outperformed the control group in terms of hemostasis duration, pain control, swelling reduction, wound healing, and postoperative complications. Similarly, in surgical treatment for benign prostatic hyperplasia, intravenous HBA shortened patients’ PT and length of hospital stay, with good safety and promising hemostatic effects.^[11]^ Although the risk of blood transfusion was identical between the HBA group and the non-HBA group (odds ratio = 1.582, 95% confidence interval = 0.552–4.538), the hemostatic advantages of HBA remained evident. In patients receiving colonic polypectomy, HBA achieved a hemostasis success rate of 99.0% and showed a shorter hemostasis time than the saline group (*P* < .001).^[20]^ Meanwhile, rebleeding and delayed bleeding rates were also significantly lower in the HBA group (*P* = .009, *P* = .009, and *P* = .048, respectively) while the baseline coagulation profiles were comparable.

In our research, we observed a trend of decreased postoperative complications in the HBA group compared to the non-HBA group, particularly in terms of lung infections and persistent air leaks. Since these differences were not statistically significant, we believe that HBA has no correlation with the occurrence of these 2 complications. Additionally, in our study, no adverse thrombotic event, and no deaths occurred within 30 days, aligning with previous research.^[11,24]^ However, it should be noted that long-term use of HBA can lead to hemocoagulase-associated hypofibrinogenemia. Ma et al^[23]^ revealed that 0.44% (8/3151) of patients using HBA experienced hemocoagulase-induced hypofibrinogenemia. Although multivariate analysis indicated that HBA was not an independent risk factor for hemocoagulase-induced hypofibrinogenemia, several studies hold opposite opinions.^[25–27]^ Nevertheless, large-scale studies are needed to determine the association between HBA and hemocoagulase-induced hypofibrinogenemia, and potential mechanisms.

Due to the manipulation of arteries and veins, vascular endothelium may be damaged during VATS. Additionally, the stapler used during surgery can irritate tissues and promote coagulation. Postoperative bed rest and pain stress further contribute to a hypercoagulable state in patients, leading to thrombus formation. In our study, D-dimer, INR, APTT, PT, and FIB levels were prolonged, while TT was decreased on the second postoperative day compared to preoperative levels, indicating that coagulation function temporarily changes after surgery.

To investigate changes in postoperative coagulation function, we used a 1:2 PSM to balance preoperative D-dimer, INR, APTT, PT, FBI, and TT levels (*P* > .05), thereby eliminating confounding factors. Regarding postoperative changes in coagulation function, we found that the TT value was longer in the HBA group compared to the non-HBA group (*P* = .006), while those values for both groups were within normal ranges (16.83 vs 15.94).

Additionally, univariate and multivariate linear regression analyses showed that HBA and preoperative TT were independent risk factors for postoperative TT, which aligns with previous findings that HBA has no impact on coagulation function.^[14,24]^ However, a systematic review and meta-analysis on cardiac surgery found that patients treated with hemocoagulase had significantly shorter postoperative PT, APTT, and TT, higher FIB levels, and similar D-dimer levels compared to control patients.^[28]^ Chen et al^[20]^ reported that postoperative PT, TT, APTT, and FIB were significantly lower in patients treated with topical HBA compared to those using metal-clip closure or electrocoagulation (*P* < .05). Regarding this discrepancy, we assumed that topical high-concentration HBA may affect systemic coagulation function through possible mechanisms, for instance, locally produced coagulation factors or platelet activation factors may circulate and causes systemic effects.

Previous studies suggest that HBA improves patient’s outcome.^[11,12,16,19,20,24]^ Li et al^[11]^ found that HBA had shorter hospital stays (6.56 ± 3.60 vs 8.20 ± 3.09 days, *P* < .01) compared to non-HBA group. Nagabhushan et al^[19]^ reported lower blood loss and drainage volume in patients using HBA during lumbar spinal fusion. Chen et al^[20]^ found that local spray of HBA after colon polyp removal improved hemostasis, shortened hemostasis time, and reduced rebleeding and late postoperative bleeding, with lower costs. Similarly, our study also showed that the HBA group had shorter hospital stays (5 vs 6 days, *P* = .001) and reduced drainage volume (400 vs 600 mL, *P* = .003), which is consistent with previous findings.^[16]^ While drainage volume is influenced by many surgical factors, the effect of HBA requires further evaluation.

This study has several strengths. Firstly, it fills the knowledge gap of the effect of intrathoracic HBA injection on VATS outcomes. To our knowledge, this is the first report to couple propensity-score-matched real-world data with peri-operative coagulation profiling, providing initial evidence that local batroxobin shortens hospital stay and reduces drainage without increasing thrombotic risk. These findings establish a foundation for dose-finding trials and blood-sparing guidelines in thoracoscopic lung surgery. Rigorous retrospective design and PSM reduce selection bias and enhance reliability.

However, this study has several limitations. As a retrospective analysis, selection bias cannot be avoided and data quality may affect results. The small sample size limits the expansion of our findings to other situations. Long-term follow-up data are not available, which limits the assessment of how HBA affects long-term prognosis. Additionally, coagulation profiles were measured only once within 24 hours after surgery, so delayed changes in fibrinogen or TT may have been missed; the fixed 10-unit dose was not compared with other regimens, leaving the optimal dose undefined; and rare adverse events could not be captured. Future research may address these limitations by increasing sample size, improving data quality, collecting long-term data, and deeply exploring coagulation parameters to fully assess HBA’s efficacy and safety.

Further validation is needed based on larger sample sizes, particularly in prospective, multicenter studies. Looking ahead, 3 lines of research are now warranted. First, a dose-finding Phase I/II trial using a 3 + 3 escalation design should compare 5, 10, and 20 units of intrapleural HBA to map the exposure-response curve for thrombin-time prolongation and fibrinogen decline, thereby establishing the minimum effective dose and the maximum tolerated dose. Second, dedicated pharmacokinetic studies with serial blood and pleural-fluid sampling (0–48 hours) using a validated chromogenic assay to quantify batroxobin activity are required to clarify local versus systemic clearance and to model optimal dosing intervals. Finally, a multicenter, double-blind, saline-controlled, non-inferiority RCT powered for hard endpoints (30-day hemostatic failure [reoperation for bleeding >200 mL within 24 hours] and thrombo-embolic events) will provide the safety and efficacy profile necessary for guideline adoption.

To sum up, intrathoracic injection of HBA shortened postoperative hospital stay and reduced drainage volume in VATS. However, it was also associated with prolonged postoperative PT. HBA may benefit patients with normal coagulation function before surgery. Further validation of the effects of intrathoracic HBA injection is needed based on larger sample sizes, particularly in prospective, multicenter studies.

## Author contributions

**Conceptualization:** Yilian Xie, Yingding Ruan.

**Data curation:** Yilian Xie, Jianwei Han, Jiajia Yang, Wei Xiong, Junsen Ye, Aiming Yang.

**Formal analysis:** Junsen Ye.

**Investigation:** Yilian Xie, Jianwei Han, Jiajia Yang, Wei Xiong, Aiming Yang.

**Methodology:** Yilian Xie, Jianwei Han, Aiming Yang.

**Supervision:** Yingding Ruan.

**Writing – original draft:** Yilian Xie.

**Writing – review & editing:** Yilian Xie, Yingding Ruan.
